# Regulation of mechanical intercellular junctions and components of the mitochondrial electron transport chain and the TCA cycle in the hypertrophic heart by pyridostigmine or trandolapril

**DOI:** 10.3389/fmolb.2026.1858563

**Published:** 2026-07-15

**Authors:** Zdenka Drastichova, Lucie Hejnova, Almos Boroš, Michal Behuliak, Jiri Novotny

**Affiliations:** 1 Department of Physiology, Faculty of Science, Charles University, Prague, Czechia; 2 Institute of Physiology, Czech Academy of Sciences, Prague, Czechia

**Keywords:** adherens junction, cardiac hypertrophy, desmosome, proteomics, pyridostigmine, sarcomere, SHR rats, trandolapril

## Abstract

The conventional approach to studying hypertension and cardiac hypertrophy primarily focuses on electrochemical signaling mediated by gap junctions. However, mechanical adherens junctions also contribute to hypertrophy through activation of β-catenin, promoting transcription of growth-inducing genes. Pyridostigmine reduces heart rate, whereas trandolapril is clinically employed as an antihypertensive agent. In this study, we investigated the impact of both drugs on the cardiac phosphoproteome of hypertensive rats (SHR) and normotensive Wistar-Kyoto (WKY) rats. Label-free LC–MS quantification was used to identify alterations in biological processes and cellular compartments. Both agents induced changes in the phosphorylation of proteins associated with adherens junctions, desmosomes, the mitochondrial electron transport chain, and metabolic pathways, each displaying distinct drug-specific patterns. Our data indicate that adaptive remodeling in hypertensive hearts is largely governed by phosphorylation-mediated regulation of the entire area composita, particularly adherens and desmosomal junctions, highlighting the crucial role of mechanical junctions in addition to electrochemical signaling. The observed ability of trandolapril to restore phosphorylation of junctional proteins, which may contribute to the suppression of cardiac hypertrophy, underscores the importance of preserving cardiomyocyte mechanical stability in the management of hypertension-related pathologies.

## 1 Introduction

Cell–cell junctions are essential for maintaining tissue integrity, mediating cellular adhesion, and enabling intercellular communication. Based on their structure and function, they are classified into communicating, anchoring, and occluding junctions. Anchoring junctions, including adherens junctions and desmosomes, mechanically link cytoskeletal filaments to the plasma membrane and contribute to cellular cohesion and mechanical stability (Campas et al., 2024; Kowalczyk and Green, 2013). Intracardiac communication is mediated either directly through physical connections between cells, such as gap junctions and tunneling nanotubes, or indirectly through the exchange of soluble factors and extracellular vesicles. Tunneling nanotubes establish cytoplasmic connections between distant cells, enabling the transfer of small molecules, signaling mediators, and even organelles. In contrast, gap junctions form intercellular channels between closely adjacent cells, allowing the direct exchange of ions and small signaling molecules and thereby facilitating rapid electrical and metabolic coupling (Zurzulo. 2021 Martins-Marques, 2021). They facilitate direct intercellular communication by enabling the transfer of ions and small molecules, such as cAMP, cGMP, and ATP, between the cytoplasmic compartments of adjacent cells (Goodenough and Paul, 2009). These structures are critical for the propagation of metabolic and electrical signals.

The organization of cell junctions in cardiomyocytes differs substantially from that observed in epithelial tissues, reflecting the distinct functional properties of these cell types. Basal epithelial cells undergo continuous proliferation, and their progeny progressively differentiate as they migrate toward the apical surface (Green and Gaudry, 2000). Consequently, epithelial junctions exhibit considerable plasticity and dynamic remodeling. In contrast, terminally differentiated cardiomyocytes display a highly specialized and stable junctional organization. Their intercellular connections are concentrated within intercalated discs (ICDs), which comprise desmosomes, adherens junctions, and gap junctions (Patel and Green, 2014). Notably, desmosomes and adherens junctions are not structurally distinct in this context but are co-localized within extended contact regions between cardiomyocytes. This hybrid junctional structure, referred to as the area composita, has been characterized using electron and immunoelectron microscopy (Franke et al., 2006; Borrmann et al., 2006). Structural and functional remodeling of intercalated discs may impair the transmission of contractile force and disrupt electrical coupling, thereby compromising both mechanical and electrical synchronization of the myocardium.

Gap junctions are composed of hexameric hemichannels formed by connexin (Cx) proteins, which assemble into plaques that facilitate intercellular exchange. Connexin 43 (Cx43), one of the most abundantly expressed connexins, is highly prevalent in both the cardiovascular and nervous systems (Zhang et al., 2023; Brooke et al., 2012; Mezzano et al., 2014). Alterations in connexin expression, phosphorylation status, and function are associated with a wide range of pathological conditions. In addition to neurological disorders, such changes have been implicated in hypertension, cardiac hypertrophy, heart failure, and arrhythmogenesis. Cx43, which is predominantly localized within intercalated discs, plays a critical role in the propagation of action potentials (Pun et al., 2022). Cx43 is subject to extensive post-translational modification, particularly phosphorylation at multiple serine and threonine residues. Several kinases, including PKA, PKC, Akt, CK1, p38 MAPK, and Src, target specific phosphosites (Yogo et al., 2006; Cooper and Lampe, 2002; Zhang et al., 2023). The phosphorylation pattern of Cx43—encompassing residues Ser325, Thr326, Ser328, Ser330, Ser364, Ser365, Ser368, Ser369, and Ser373—critically determines its structural conformation, channel activity, protein interactions, and subcellular localization (Pun et al., 2022; Cooper and Lampe, 2002; Solan et al., 2007; Yogo et al., 2006). Notably, PKC-mediated phosphorylation of Ser368 reduces gap junction permeability and promotes lateral redistribution of Cx43 to the lateral membrane of cardiomyocytes (Ek-Vitorin et al., 2006; Lampe et al., 2000; Jabr et al., 2016; Zhong et al., 2022).

Adherens junctions are composed of cadherins, armadillo family proteins, and cytoskeletal adaptor proteins. The cytoplasmic domain of cadherins interacts with the catenin complex, including β-catenin, α-catenin, and p120-catenin (also known as δ-catenin 1) (Campas et al., 2024). Additional regulatory proteins, such as afadin and vinculin, have been identified as key modulators of actin filament anchorage to adherens junctions (Bejar-Padilla et al., 2022; Sakakibara et al., 2020; Noethel et al., 2018). α-Catenin and vinculin cooperate to link F-actin to the cadherin–catenin complex, thereby stabilizing the junction and reducing its sensitivity to fluctuations in intercellular tension (Bejar-Padilla et al., 2022). While α- and β-catenins mediate the physical linkage between cadherins and actin filaments, p120-catenin regulates cadherin stability and turnover within the junction. Specifically, the Armadillo repeats 1–7 of p120-catenin interact directly with the C-terminal juxtamembrane domain of cadherins (Reynolds and Roczniak-Ferguson, 2004; Horikawa and Takeichi, 2001).

Desmosomes represent the third major type of junction within intercalated discs. They are composed of desmosomal cadherins, armadillo proteins, and plakin family proteins. The desmosomal core, consisting of desmoglein and desmocollin, is embedded in the plasma membrane and interacts with corresponding proteins on adjacent cells. The intracellular domains of these cadherins are linked to the cytoskeleton through adaptor proteins, including desmoplakin, plakophilin, and plakoglobin, thereby providing mechanical resilience (Lessey et al., 2022; Patel and Green, 2014).

Alterations in the structure of the intercellular cleft, mutations in genes encoding intercalated disc proteins, or disruption of cardiac tissue architecture can impair electrochemical signaling. These changes increase the susceptibility to arrhythmias and cardiomyopathies (Patel and Green, 2014; Chkourko et al., 2012). Importantly, structural and functional remodeling of intercalated disc junctions is generally considered a consequence, rather than a primary cause, of hypertension. Within this context, gap junctions have been the primary focus of investigation. Chronic hypertension has been shown to reduce Cx43 expression and alter its phosphorylation pattern, resulting in lateral redistribution of Cx43 and a decrease in functional intercellular coupling (Huang et al., 2020; Szeiffova Bacova et al., 2023; Fialova et al., 2008).

Gap junction conductivity is regulated by intracellular kinases through modulation of the phosphorylation state of Cx43. One of the key regulatory pathways involves acetylcholine-mediated activation of M2 muscarinic receptors. Acetylcholine is rapidly degraded by acetylcholinesterase (AChE), thereby terminating synaptic transmission (Khuanjing et al., 2020). Pharmacological inhibition of AChE by pyridostigmine has been shown to attenuate cardiac hypertrophy, which develops as a compensatory response to chronic systemic arterial hypertension (Lataro et al., 2013). This pathological condition is characterized by enhanced sympathetic activity, reduced parasympathetic tone, activation of the renin–angiotensin system (RAS), increased production of reactive oxygen species (ROS), and inflammation (Hirooka, 2020). Pyridostigmine exerts parasympathomimetic effects, partly through activation of M3 muscarinic receptors and subsequent nitric oxide (NO) release (Cavalcante et al., 2021). Furthermore, pyridostigmine has been reported to prevent the decrease in Cx43 expression following myocardial infarction (Santos-Almeida et al., 2015).

An alternative therapeutic strategy for hypertension involves modulation of the RAS. Trandolapril, an angiotensin-converting enzyme (ACE) inhibitor, effectively reduces the formation of angiotensin II (Peters et al., 1998). It has been shown to modulate connexin expression by reducing levels of Cx43 and Cx40 while increasing PKCε expression, thereby influencing connexin phosphorylation (Sykora et al., 2025; Cruciani and Mikalsen, 2002). However, the effects of these pharmacological agents on other types of intercellular junctions have not been investigated.

In our previous study, trandolapril significantly reduced mean arterial pressure in both Wistar–Kyoto (WKY) and spontaneously hypertensive (SHR) rats and attenuated cardiac hypertrophy in SHR animals. SHR rats exhibited alterations in cholinergic signaling and cAMP pathways compared to WKY controls (Hejnova et al., 2024). Although both pyridostigmine and trandolapril influence the cholinergic system, their mechanisms of action differ. The aim of the present study was therefore to determine whether the effects of pyridostigmine and trandolapril on cardiac function are associated with changes in the regulation of intercellular junction proteins. To this end, label-free bottom-up mass spectrometry-based proteomic and phosphoproteomic approaches were employed to quantify alterations in protein expression and phosphorylation within intercellular junction components in the left ventricle of SHR rats. In parallel, the expression and phosphorylation of proteins associated with the mitochondrial electron transport chain and the TCA cycle were also assessed a key components modulating cardiomyocyte bioenergetics and function.

## 2 Materials and methods

### 2.1 Materials

Pyridostigmine and trandolapril were purchased from Sigma-Aldrich (St. Louis, MO, United States) and Mylan (Canonsburg, PA, United States), respectively. All other chemicals were purchased from Sigma-Aldrich (St. Louis, MO, United States).

### 2.2 Animals and treatment

Experiments were conducted using groups of age-matched 4- to 5 month-old Wistar–Kyoto (WKY) and spontaneously hypertensive (SHR) rats. At this age, SHR rats exhibit a well-established phenotype characterized by systemic hypertension and cardiac hypertrophy. All animals were housed under standard laboratory conditions, including a controlled ambient temperature of 23 °C ± 1 °C, a 12 h light–dark cycle, and free access to a standard rodent diet and water. Animals received either pyridostigmine (25 mg/kg/day) or trandolapril (1 mg/kg/day), administered via drinking water over a period of 8 weeks. Following treatment, the animals were weighed and deeply anesthetized with isoflurane (5% in air) and subsequently euthanized by decapitation for tissue collection. All experimental procedures were approved by the Committee for Animal Care at the Institute of Physiology, Czech Academy of Sciences, and were conducted in accordance with the European Convention on Animal Protection, as well as relevant guidelines for the use of laboratory animals.

### 2.3 Specimen collection and processing

Following treatment, animals were anesthetized with isoflurane, weighed, and subsequently euthanized. Hearts were excised and weighed, and tissue samples from the left ventricle were collected into Eppendorf tubes, snap-frozen in liquid nitrogen, and stored at −80 °C until further analysis. Left ventricular samples from eight animals per experimental group were used for biochemical investigations. Approximately 200 mg of heart tissue was diluted 1:4 in phosphate-buffered saline (PBS) and homogenized twice for 10 s using an Ultra-Turrax homogenizer. A second homogenization step was performed for 2 min at 1,200 rpm using a motor-driven glass–Teflon homogenizer. Individual homogenates from each experimental group were pooled to generate three biological replicates, each comprising two or three samples per group. Pooled homogenates were further diluted 1:1 with PBS. For proteomic analysis, 500 µL of each homogenate was mixed 1:1 with 100 mM triethylammonium bicarbonate (TEAB) buffer containing 2% (w/v) sodium deoxycholate (SDC). Samples were sonicated three times for 10 s at 40% amplitude (Bandelin sonicator), followed by centrifugation at 14,000 rpm for 10 min at 4 °C. A 100 µL aliquot of the supernatant was adjusted to a final protein concentration of 1 μg/μL using 50 mM TEAB buffer containing 1% SDC and snap-frozen in liquid nitrogen. Protein concentration was determined using the bicinchoninic acid (BCA) assay. For phosphoproteomic analysis, 500 µL of each homogenate was lysed by heating at 95 °C for 10 min in 100 mM Tris-HCl, pH 8.5, containing 2% sodium deoxycholate. Chloroacetamide and Tris (2-carboxyethyl)phosphine were added to final concentrations of 40 mM and 10 mM, respectively. Protein concentration was determined using the BCA protein assay kit (Thermo), and 250 µg of protein per sample was used for mass spectrometry preparation. Proteins were digested with 5 µg trypsin per sample at 37 °C overnight. Phosphopeptides were subsequently enriched using TiO_2_ according to the method described by Humphrey et al. (2018).

### 2.4 Proteomic analysis by nLC-MS2

LC/MS analysis of heart samples was performed using nano-reversed-phase chromatography on a PepMap C18 column (EASY-Spray, 50 cm × 75 μm ID, 2 μm particle size, 100 Å pore size). Mobile phase A consisted of 0.1% formic acid in water, while mobile phase B contained 0.1% formic acid in acetonitrile. Samples were initially loaded onto a C18 PepMap100 trap column (5 μm particle size, 300 μm × 5 mm; Thermo Scientific, Waltham, MA, United States) using a loading buffer comprising 2% acetonitrile and 0.1% trifluoroacetic acid in water. Sample loading was conducted at a flow rate of 18 μL/min for 4 min. Peptides were eluted using a linear gradient of mobile phase B from 2% to 35%. Eluted peptide cations were ionized by electrospray ionization and analyzed on a Thermo Scientific Orbitrap Fusion mass spectrometer (Q-OT-qIT configuration). Survey scans of peptide precursors were acquired over an m/z range of 350–1,400 at a resolution of 120,000 (at m/z 200) with an ion target of 1 × 10^6^. Tandem MS (MS/MS) analysis was performed using quadrupole isolation with a 1.5 Th window, followed by higher-energy collisional dissociation (HCD) at a normalized collision energy of 35 and rapid scan analysis in the ion trap. The maximum injection time was set to 150 ms, with an MS2 ion target of 1 × 10^4^. Only precursor ions with charge states between 2 and 6 were selected for fragmentation. Dynamic exclusion was enabled for 30 s, with a mass tolerance of 10 ppm for the selected precursor and its isotopes. The cycle time was set to 2 s, and monoisotopic precursor selection was applied throughout the analysis.

### 2.5 Data analysis

To assess protein expression and phosphorylation, six experimental groups were analyzed using label-free LC-MS. Samples were obtained from the left ventricles of control WKY rats (WC), WKY rats treated with pyridostigmine (WP) or trandolapril (WT), control SHR rats (SC), and SHR rats treated with pyridostigmine (SP) or trandolapril (ST). Signal intensities from triplicate samples were summarized as median values and compared across seven pairwise comparisons: SC/WC, WP/WC, WT/WC, SP/SC, ST/SC, SP/WP, and ST/WT. Raw L/MS data were processed and quantified using MaxQuant software. The false discovery rate (FDR) was set at 1% for both peptide and protein identifications, with a minimum peptide length of seven amino acids. MS/MS spectra were searched against the *Rattus norvegicus* database using the Andromeda search engine. Enzyme specificity was set to cleavage C-terminal to arginine and lysine residues, including cleavage before proline, allowing up to two missed cleavages. Carbamidomethylation of cysteine was specified as a fixed modification, whereas protein N-terminal acetylation and methionine oxidation were included as variable modifications. The “match between runs” feature in MaxQuant was enabled to transfer peptide identifications across LC–MS/MS runs based on accurate mass and retention time (maximum deviation of 0.7 min). This feature was also applied for label-free quantification (LFQ). Protein quantification was performed using the MaxQuant LFQ algorithm, and downstream data analysis was conducted in Perseus. Proteins exhibiting qualitative or quantitative differences between groups in a pairwise comparison were considered differentially altered. A qualitative change was defined as the presence or absence of a protein in one group, consistently observed in at least two of the three biological replicates. A quantitative change was defined as a minimum twofold difference in protein expression between groups, with the protein detected in at least two of three biological replicates. Phosphoproteins meeting these criteria were subjected to functional annotation using the Database for Annotation, Visualization, and Integrated Discovery (DAVID). Gene Ontology (GO) biological processes related to intercellular junctions, cytoskeleton organization, and cardiac function were subsequently selected, and their enrichment (−log p value) was visualized graphically. Figures were generated using BioRender.com.

## 3 Results

### 3.1 Altered expression of proteins related to intercellular junctions

The gene ontology (GO) biological processes enriched in differentially abundant proteins in the left ventricles of SHR rats treated with pyridostigmine or trandolapril were previously reported (Hejnova et al., 2024). Both drugs affected the expression of proteins involved in translation, proteasomal degradation, and RNA regulation. GO analysis performed using DAVID revealed enrichment of cell adhesion processes in the SC/WC proteomic comparison. Endothelial adhesion molecules, including ESAM and MCAM, together with membrane adhesive receptors CD9 and CD36, were downregulated in control SHR rats compared to control WKY rats (Table 1; Supplementary Table S1). A mild decrease in GJA1 (Cx43) expression was also observed in the SC/WC comparison (Table 1; Supplementary Table S1). These findings indicate that the primary differences between the two strains are related to endothelial regulation within vessels rather than structural rearrangement of intercellular junctions between cardiomyocytes. Treatment with either pyridostigmine or trandolapril did not significantly affect the expression of ESAM, MCAM, CD9, CD36, or GJA1 in SHR hearts, suggesting that these drugs do not substantially influence electrochemical signal transmission between cardiomyocytes in this model of hypertension.

**TABLE 1:** List of differentially expressed proteins related to cell adhesion and gap junctions identified in the pairwise comparisons SC/WC, SP/SC, and ST/SC.

UniProt ID	Gene ID	Gene name	SC/WC	SP/SC	ST/SC
P08050	Gja1	Gap junction α-1 protein	-2.2	-	-
Q07969	Cd36	Platelet glycoprotein 4	-4.6	-	-
P40241	Cd9	CD9 antigen	-2.7	-	-
Q6AYD4	Esam	Endothelial cell-selective adhesion molecule	WC	-	-
Q9EPF2	Mcam	Cell surface glycoprotein MUC18	WC	-	-

SC, control SHR rats; SP, SHR rats treated with pyridostigmine; ST, SHR rats treated with trandolapril; WC, control Wistar Kyoto rats.

### 3.2 Altered phosphorylation of proteins related to intercellular junctions and sarcomeres

**FIGURE 1:**
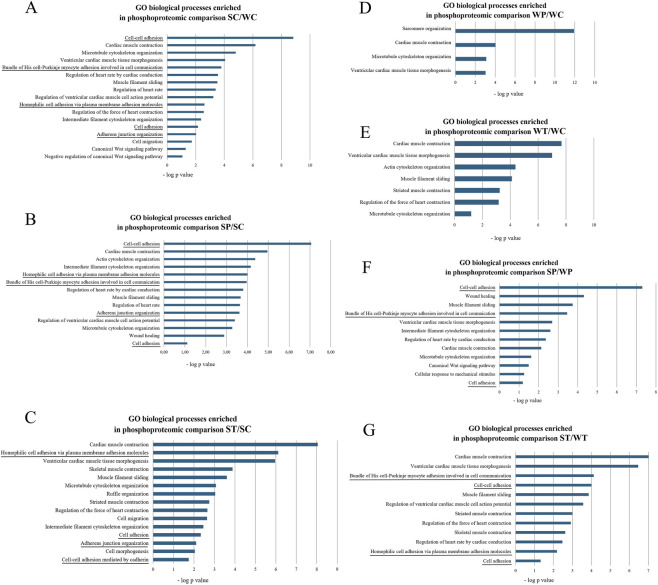
Gene ontology analysis of enriched biological processes by the DAVID tool. The negative -log_10_ transformed *p*-values of enriched biological processes are indicated for comparisons SC/WC **(A)**, SP/SC **(B)**, ST/SC **(C)**, WP/WC **(D)**, WT/WC **(E)**, SP/WP **(F)** and ST/WT **(G)**.

GO analysis revealed that biological processes related to cell adhesion, cytoskeletal and junction organization, and cardiac function were significantly enriched among differentially phosphorylated proteins in comparisons SC/WC, SP/SC, and ST/SC (Figures 1A–C). These processes were absent in WP/WC and WT/WC comparisons (Figures 1D,E), indicating that pyridostigmine and trandolapril do not affect phosphorylation-mediated regulation of intercellular junctions in Wistar-Kyoto rats. The differences in response between SHR and WKY rats were mainly observed as distinct phosphorylation patterns in components of intercellular junctions (Figures 1F,G).

**FIGURE 2:**
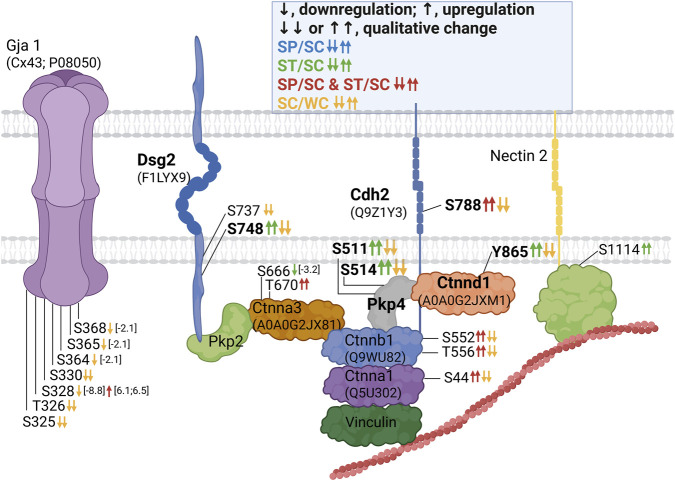
Alterations in phosphorylation of proteins related to gap and adherens juctions. Proteins with altered phosphorylation in comparisons of control SHR versus WKY rats (SC/WC), pyridostigmine-treated SHR versus control SHR rats (SP/SC), and trandolapril-treated SHR versus control SHR rats (ST/SC) are labeled with gene names and protein identifiers from the UniProt database (www.uniprot.org). Phosphosites are annotated using standard amino acid abbreviations (S, serine; T, threonine; Y, tyrosine) followed by their position within the protein sequence. Small upward and downward arrows indicate increased and decreased phosphorylation, respectively. Pairwise experimental comparisons are distinguished by color (SP/SC, blue; ST/SC, green; concurrent alterations in SP/SC and ST/SC, red; SC/WC, yellow). Protein associations and interactions are indicated by arrows denoting stimulatory or inhibitory effects and are organized according to Palatinus et al. (2012), Sun et al. (2014), Zhao et al. (2019), Rampazzo et al. (2014), Li (2014), Sheikh et al. (2009). Abbreviations: Cdh2, cadherin-2/N-cadherin; Ctnna1, catenin α-1; Ctnna3, catenin α-3; Ctnnb1, catenin β-1; Ctnnd1, catenin δ-1; Dsg2, desmoglein-2; Gja1, gap junction α-1 protein/connexin-43; Mllt4, afadin; Pkp2, plakophilin-2; Pkp4, plakophilin-4.

A closer examination of the altered proteins revealed that phosphorylation changes were primarily observed in components of desmosomes and adherens junctions. Phosphorylation of Gja1 (Cx43), the major gap junction protein, was decreased at seven phosphosites (Ser325, Thr326, Ser328, Ser330, Ser364, Ser365, and Ser368) in control SHR rats compared to WKY rats (Supplementary Table S2; Figure 2). This hypophosphorylation correlated with the modest decrease in Gja1 expression. Only Ser328 phosphorylation was restored by both pyridostigmine and trandolapril (Supplementary Table S2; Figure 2), suggesting limited reversal of pathological conductivity disturbances in hypertrophied hearts.

Altered phosphorylation was also observed in components of adherens junctions. Cadherin-2 (Cdh2, N-cadherin) was dephosphorylated at Ser788 in SHR compared to WKY rats (Supplementary Table S2; Figure 2). Interacting catenins exhibited similar changes: Tyr865 in Ctnnd1, Ser552 and Ser556 in Ctnnb1, and Ser44 in Ctnna1. Phosphorylation of plakophilin-4 (Ser511 and Ser514) was also decreased. Except for Tyr865 in Ctnnd1, phosphorylated only by trandolapril, both substances restored phosphorylation of these sites to WKY levels (Supplementary Table S2; Figure 2). Additionally, trandolapril hyperphosphorylated Mllt4 (afadin) at Ser1114, and both substances increased phosphorylation at Thr670 of Ctnna1 (α-catenin), while trandolapril attenuated phosphorylation at Ser666 (Figure 2; Supplementary Table S2).

**FIGURE 3:**
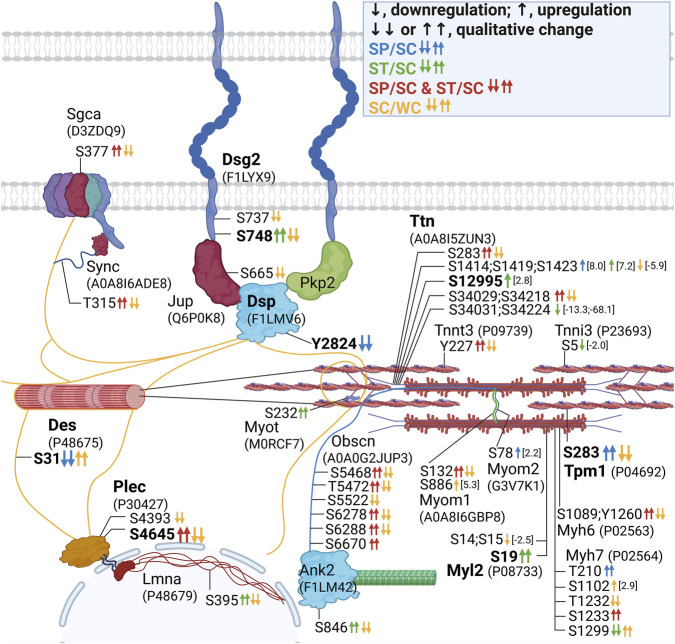
Alterations in phosphorylation of proteins related to desmosomes. The description and layout of the figure are the same as in Figure 2. Protein associations and interactions are organized according to Zhao et al. (2019), Rampazzo et al. (2014), Li (2014), Sheikh et al. (2009), Pernigo et al. (2017), Berika and Garrod (2014), Goldmann (2018), Lange et al. (2020), Kostan et al. (2021), Lin et al. (2021), Mohammed and Chidkey (2021), Su et al. (2022). Abbreviations: Ank2, ankyrin-2; Des, desmin; Dsg2, desmoglein-2; Dsp, desmoplakin; Jup, junction plakoglobin; Lmna, lamin A; Myh6, myosin-6; Myh7, myosin-7; Myl2, myosin regulatory light chain 2; Myom1, myomesin-1; Myom2, myomesin-2; Myot, myotilin; Obscn, obscurin; Pkp2, plakophilin-2; Plec, plectin; Sgca, α-sarcoglykan; Sync, syncoilin; Tnni3, troponin I, cardiac muscle; Tnnt3, troponin T, fast skeletal muscle; Tpm1, tropomyosin-1; Ttn, titin.

Interestingly, several desmosomal proteins and associated sarcomeric components were dephosphorylated in SHR hearts compared with WKY controls. In SHR hearts, desmoglein-2 (Dsg2) was dephosphorylated at Ser737 and Ser748, and junction plakoglobin (Jup) at Ser665 (Figure 3; Supplementary Table S2). Trandolapril restored phosphorylation at Ser748 in Dsg2. Desmoplakin (Dsp) was dephosphorylated following pyridostigmine treatment at Tyr2824 (Figure 3; Supplementary Table S2).

Desmin, an intermediate filament protein that interacts with desmoplakin, was hyperphosphorylated at Ser31 in control SHR rats compared with control WKY rats, and pyridostigmine reversed this phosphorylation state (Figure 3; Supplementary Table S2). Trandolapril reduced this phosphorylation less efficiently, showing this effect in only two of three replicates (Supplementary Table S2). Desmin interacts with multiple cellular compartments, several of which were found to be differentially phosphorylated (Figure 3). α-Sarcoglycan (Sgca) and syncoilin (Sync), which associate with the dystrophin–glycoprotein complex in the sarcolemma of cardiomyocytes, were dephosphorylated in control SHR rats compared with WKY controls, and their phosphorylation states were restored by both treatments (Figure 3; Supplementary Table S2). Desmin also interacts with plectin (Plec), which links it to the nuclear membrane and lamin A (Lmna) via the LINC complex (West et al., 2023). Both proteins were dephosphorylated at several phosphosites in control SHR rats compared with control WKY rats, with phosphorylation restored by trandolapril in Lmna and by both treatments in Plec (Figure 3; Supplementary Table S2).

Finally, structural proteins of the sarcomere were also affected. In thick filaments, both major myosin isoforms, Myh6 and Myh7, were differentially phosphorylated in SHR rats compared with WKY controls (Figure 3; Supplementary Table S2). The phosphorylation state of Myh6 was fully restored by both treatments, whereas Myh7 exhibited distinct phosphorylation patterns depending on treatment with pyridostigmine or trandolapril (Figure 3; Supplementary Table S2). The regulatory myosin light chain-2 (Myl2, MLC-2) was differentially phosphorylated at three serine residues. Ser14 and Ser15 were mildly hypophosphorylated in control SHR rats compared with control WKY rats, while trandolapril induced hyperphosphorylation at Ser19 in SHR rats (Figure 3; Supplementary Table S2).

Although the phosphorylation state of actin remained unchanged, the tropomyosin–troponin complex exhibited differential phosphorylation under various conditions (Figure 3). Phosphorylation at Ser283 in tropomyosin α-1 (Tpm1), absent in control SHR rats compared with control WKY rats, was restored by both treatments (Figure 3; Supplementary Table S2). Similarly, phosphorylation at Tyr227 in troponin T (Tnnt3), which was abrogated in control SHR rats, was recovered by both substances (Figure 3; Supplementary Table S2). Troponin I (Tnni3) displayed mild dephosphorylation following trandolapril treatment (Figure 3; Supplementary Table S2). Myotilin (Myot), which interacts with F-actin and α-actinin to stabilize Z-discs, was hyperphosphorylated at Ser232 by trandolapril (Figure 3; Supplementary Table S2). Consequently, phosphorylation of the M-band proteins, including myomesins, was also modulated. Both myomesins (Myom1 and Myom2), which form the M-band and link thick filaments (Pernigo et al., 2017), were differentially phosphorylated in control SHR versus WKY rats. While the abrogated phosphorylation of Myom1 was partially restored by both treatments, Myom2 was mildly hyperphosphorylated at Ser78 exclusively by pyridostigmine (Figure 3; Supplementary Table S2). Titin (Ttn), a large structural protein interacting with numerous sarcomeric components, was also markedly affected, showing differential phosphorylation at multiple phosphosites (Figure 3; Supplementary Table S2). Both treatments restored phosphorylation at Ser283, located within the Z-disc–binding region and dephosphorylated in control SHR rats compared with control WKY rats (Figure 3; Supplementary Tables S2, S3). A similar pattern was observed in the phosphosite cluster comprising Ser1414, Ser1419, and Ser1423, localized in a disordered region following the fifth Ig domain (Figure 3; Supplementary Tables S2, S3). Interestingly, phosphorylation at Ser12995 was regulated independently of rat strain and was mildly increased by trandolapril (Figure 3; Supplementary Table S2). This site corresponds to human Ser12022 within the PEVK region (Supplementary Table S3) and is known to be phosphorylated by CaMKIIδ and PKCα (Mikhailova et al., 2024). Finally, a cluster of phosphosites, including Ser34024, Ser34029, Ser34031, Ser34218, and Ser34224, is located in the region following the kinase domain and three Ig domains. In SHR rats, phosphorylation at some of these sites was restored by both treatments, whereas others were further dephosphorylated specifically by trandolapril (Figure 3; Supplementary Table S2). Obscurin (Obscn), a large structural protein that interacts with titin and myomesin (Pernigo et al., 2017), was markedly dephosphorylated at several phosphosites located within disordered regions in the central part of the molecule in control SHR rats compared with control WKY rats (Figure 3; Supplementary Tables S2, S3). Both treatments restored the phosphorylation state, with hyperphosphorylation observed at Ser6670 within the PH domain (Figure 3; Supplementary Tables S2, S3). Ankyrin 2 (Ank2), which links obscurin to microtubules, was dephosphorylated at Ser846, located between the ankyrin repeat and ZU5 domains, in control SHR rats. Trandolapril restored phosphorylation at this site (Figure 3; Supplementary Tables S2, S3).

Overall, these results indicate that phosphorylation patterns of adherens junctions, desmosomes, and sarcomeric or structural proteins are dysregulated in hypertensive SHR hearts. Both pyridostigmine and trandolapril partially restored the phosphorylation state, with trandolapril generally exerting more pronounced corrective effects, and additional compound-specific effects were observed at selected phosphosites.

### 3.3 Altered expression and phosphorylation of proteins related to electron transport chain and oxidative phosphorylation

**FIGURE 4:**
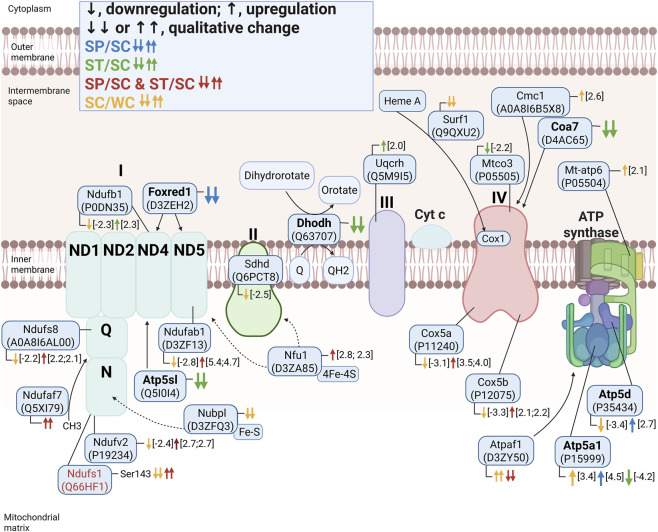
Alterations in expression and phosphorylation of proteins related to the mitochondrial electron transport chain. Proteins with altered expression (black) or phosphorylation (red) in comparisons of control SHR versus WKY rats (SC/WC), pyridostigmine-treated SHR versus control SHR rats (SP/SC), and trandolapril-treated SHR versus control SHR rats (ST/SC) are labeled with gene names and UniProt protein identifiers (www.uniprot.org). The description and layout of the figure are identical to those in Figure 2. Protein associations and interactions are indicated by arrows denoting stimulatory or inhibitory effects and are organized according to Hock et al. (2020). Abbreviations: Atp5a1, ATP synthase subunit α; Atp5d, ATP synthase subunit δ; Atp5sl, Distal membrane-arm assembly complex protein 2; Atpaf1, ATP synthase mitochondrial F1 complex assembly factor 1; Cmc1, COX assembly mitochondrial protein; Coa7, Cytochrome c oxidase assembly factor 7; Cox5a, Cytochrome c oxidase subunit 5A; Cox5b, Cytochrome c oxidase subunit 5B; Dhodh, Dihydroorotate dehydrogenase (quinone); Foxred1, FAD-dependent oxidoreductase domain-containing 1; Mtco3, Cytochrome c oxidase subunit 3; Mt-atp6, ATP synthase subunit a; Ndufab1, Acyl carrier protein; Ndufaf7, Protein arginine methyltransferase NDUFAF7; Ndufb1, NADH dehydrogenase (ubiquinone) 1β subcomplex subunit 1; Ndufs1, NADH-ubiquinone oxidoreductase 75 kDa subunit; Ndufs8, NADH dehydrogenase (ubiquinone) iron-sulfur protein 8; Ndufv2, NADH dehydrogenase (ubiquinone) flavoprotein 2; Nfu1, NFU1 iron-sulfur cluster scaffold homolog; Nubpl, NUBP iron-sulfur cluster assembly factor-like; Q, Coenzyme Q; QH2, Reduced coenzyme Q; Sdhd, Succinate dehydrogenase (ubiquinone) cytochrome b small subunit; Surf1, Surfeit locus protein 1; Uqcrh, Cytochrome b-c1 complex subunit 6.

Numerous mitochondrial proteins involved in electron transport and proton translocation were differentially expressed or phosphorylated in SHR rats compared to WKY controls, and following treatment with pyridostigmine or trandolapril (Figure 4; Supplementary Tables S1, S2). Both substances primarily modulated protein expression, with phosphorylation changes observed less frequently, particularly in subunits of mitochondrial respiratory chain complexes I, II, IV, and V, as well as in factors involved in complex assembly.

Subunits of complex I, including Ndufs8, Ndufv2, and Ndufab1, were decreased in control SHR rats compared to WKY rats and restored by both pyridostigmine and trandolapril (Figure 4; Supplementary Table S3). Ndufb1 was decreased in SHR hearts and selectively elevated by trandolapril. Phosphorylation of Ndufs1 at Ser143 was differentially regulated under experimental conditions (Figure 4; Supplementary Table S2), though its functional implications remain unknown. Several assembly factors for complex I were also affected. Atp5sl and Foxred1, which are associated with a near-complete ND4 module (Hock et al., 2020), were decreased by trandolapril and pyridostigmine, respectively (Figure 4; Supplementary Table S1). Nubpl levels were decreased in SHR hearts compared to WKY controls, while Ndufaf7, which mediates dimethylation of an arginine in Ndufs2, was increased by both drugs (Figure 4; Supplementary Table S2). Chaperone Nfu1, responsible for [4Fe-4S] cluster incorporation into complexes I and II, was mildly increased following either treatment (Figure 4; Supplementary Table S2). The complex II subunit Sdhd was slightly decreased in SHR hearts and further affected by trandolapril. Uqcrh, a component of complex III, showed a mild increase in expression following trandolapril treatment (Figure 4; Supplementary Table S1)). Within complex IV, the levels of Cox5a located in Cox1 module, Cox5b located in Cox2 module, and the assembly factor Surf1, delivering heme A to Cox1 (Hock et al., 2020), were decreased in control SHR to WKY rats (Figure 4). Both drugs restored the levels of Cox5a and Cox5b in SHR rats (Figure 4; Supplementary Table S1). The level of Mtco3 subunit located in the Cox3 module was mildly decreased by trandolapril (Figure 4; Supplementary Table S1). The levels of assembly factors of complex IV, Cmc1 and Coa7, were differentially expressed under different conditions (Figure 4).

Subunits Mt-Atp6 (FO unit) and Atp5a1 and Atp5d (F1 unit) were differentially expressed in SHR hearts relative to WKY rats, with both drugs affecting only the F1 subunits. The chaperone Atpaf1, elevated in SHR hearts, was restored to WKY levels by both treatments (Figure 4; Supplementary Table S1).

These results indicate that both pyridostigmine and trandolapril partially normalize the expression of key subunits and assembly factors of the electron transport chain, particularly in complexes I, IV, and F1 of ATP synthase, suggesting a potential recovery of mitochondrial bioenergetic function in hypertensive hearts.

### 3.4 Altered expression and phosphorylation of proteins related to the electron transport chain and oxidative phosphorylation

**FIGURE 5:**
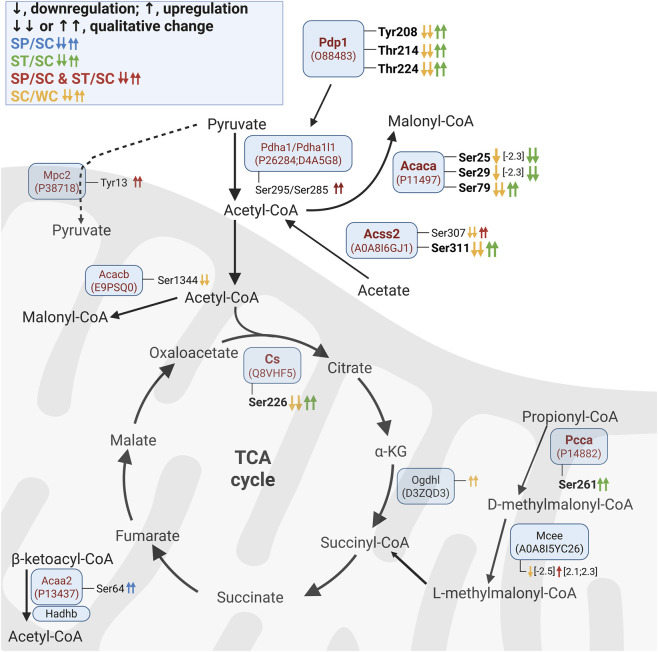
Alterations in expression and phosphorylation of proteins related to mitochondrial metabolic pathways. The description and layout of the figure are identical to those in Figure 4. Protein associations and interactions are organized according to Arnold and Finley (2023) and Houten et al. (2016). Abbreviations: α-KG, α-ketoglutarate; Acaa2, 3-ketoacyl-CoA thiolase; Acaca, Acetyl-CoA carboxylase 1; Acacb, Acetyl CoA carboxylase; Acss2, Propionate—CoA ligase; Cs, Citrate synthase; Hadhb, Trifunctional enzyme subunit β; Mcee, Methylmalonyl CoA epimerase; Mdh2, Malate dehydrogenase 2; Mpc2, Mitochondrial pyruvate carrier 2; Ogdhl, 2-oxoglutarate dehydrogenase-like; Pcca, Propionyl-CoA carboxylase α chain; Pdha/Pdha1l1, Pyruvate dehydrogenase E1 component subunit α; Pdp1, Pyruvate dehydrogenase (acetyl-transferring))-phosphatase 1.

Several enzymes involved in the TCA cycle and acetyl-CoA metabolism were differentially regulated in SHR rats compared to WKY controls, as well as in response to pyridostigmine or trandolapril treatment (Figure 5; Supplementary Tables S1, S2). Cytosolic acetyl-CoA carboxylase (Acaca) was significantly hypophosphorylated at Ser25, Ser29, and Ser79 in SHR rats relative to WKY controls. Phosphorylation at Ser79 was restored by both trandolapril, whereas Ser25 and Ser29 were fully dephosphorylated by this drug (Figure 5; Supplementary Table S2). Mitochondrial acetyl-CoA carboxylase (Acacb) exhibited hypophosphorylation at Ser1344 in SHR hearts compared to WKY controls. Acss2, responsible for converting acetate to acetyl-CoA, was dephosphorylated at Ser307 and Ser311 in SHR hearts, with trandolapril showing greater efficiency than pyridostigmine in restoring phosphorylation (Figure 5; Supplementary Table S2).

Pyruvate dehydrogenase complex subunits Pdha1 and Pdha1l1 were hyperphosphorylated at Ser295/Ser285 in SHR rats by both inhibitors (Figure 5; Supplementary Table S2). Pyruvate dehydrogenase phosphatase 1 (Pdp1) was hypophosphorylated at Tyr208, Thr214, and Thr224 in SHR hearts, and phosphorylation at these sites was restored by both drugs (Figure 5; Supplementary Table S2). The mitochondrial pyruvate carrier 2 (Mpc2) was hyperphosphorylated at Ser13 in SHR rats following both treatments (Figure 5; Supplementary Table S2).

Several enzymes of the TCA cycle were also affected. Citrate synthase (Cs) was hyperphosphorylated at Ser226 by trandolapril in SHR rats (Figure 5; Supplementary Table S2). The expression of the mitochondrial 2-oxoglutarate dehydrogenase-like enzyme (Ogdhl) was elevated in SHR hearts compared to WKY controls, and succinyl-CoA production—derived from propionyl-CoA via propionyl-CoA carboxylase (Pcca) and methylmalonyl-CoA epimerase (Mcee)—was differentially affected by both inhibitors (Figure 5; Supplementary Tables S1, S2).

Within β-oxidation, only 3-ketoacyl-CoA thiolase (Acaa2) exhibited hyperphosphorylation at Ser64 in SHR rats in response to both treatments (Figure 5 Supplementary Table S2).

## 4 Discussion

Hypertension is associated with left ventricular hypertrophy as a consequence of elevated systemic pressure. Consistent with this, we previously observed an increased heart-weight-to-body-weight ratio in hypertensive SHR rats compared with normotensive WKY rats and demonstrated that trandolapril more effectively than pyridostigmine attenuated this ratio and reduced blood pressure (Hejnova et al., 2024). Building on these findings, the present study shows that both pyridostigmine and trandolapril modulate the phosphorylation of intercellular junction proteins, cytoskeletal filaments, and sarcomeric components in hypertensive SHR hearts, without significantly altering their total protein expression. Our results support the concept that several sarcomeric proteins, as well as Cx43, may exhibit reduced phosphorylation at specific phosphosites during hypertensive cardiac hypertrophy. Notably, hypertension is associated with altered phosphorylation of adherens junction and desmosomal proteins, whereas the phosphorylation state of Cx43 remains largely unaffected by drug treatment.

In hypertensive SHR rats, all three serine residues of Cx43 (Ser325, Ser328, Ser330) were dephosphorylated compared with normotensive WKY rats, accompanied by a mild reduction in Ser368 phosphorylation and a slight decrease in total Cx43 levels. Both pyridostigmine and trandolapril restored phosphorylation at Ser328 but had no effect on other Cx43 phosphosites, suggesting that the anti-hypertrophic action of trandolapril is unlikely to be mediated through restoration of Cx43 phosphorylation or gap junction function. Previous studies have shown that Cx43 lateralization and reduced Ser368 phosphorylation are associated with impaired electrical conduction in hypertrophic hearts (Jin et al., 2010; Andelova et al., 2024; Andelova et al., 2022).

**FIGURE 6:**
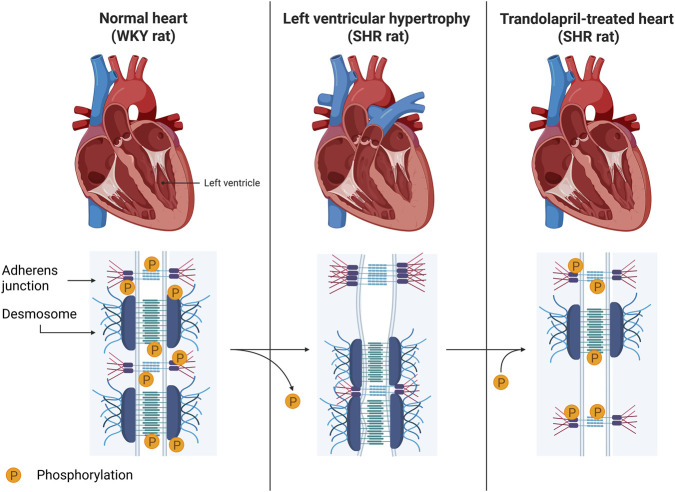
The effect of trandolapril on morphological and molecular changes in intercalated discs during hypertensive left ventricular hypertrophy can be summarized as follows: in the normal heart, intercalated discs—comprising adherens junctions and desmosomes—are thin, symmetric, and highly organized, with components of mechanical junctions predominantly in a phosphorylated state. In contrast, cardiac hypertrophy is characterized by thicker, irregularly shaped intercalated discs and by dephosphorylation of junctional components. The primary effect of trandolapril in hypertensive SHR hearts is the attenuation of hypertrophy and the restoration of the phosphorylation state of mechanical junction proteins.

Desmosomes and adherens junctions have been implicated in multiple cardiac diseases. Arrhythmogenic right ventricular cardiomyopathy/dysplasia, a life-threatening arrhythmia syndrome, is caused by mutations in genes encoding desmosomal proteins and is associated with intercalated disc remodeling, including adherens junction reorganization (Thiene et al., 2007). Similarly, desmosomal gene mutations are frequently observed in patients with idiopathic dilated cardiomyopathy (Garcia-Pavia et al., 2011; Elliott et al., 2010). Naxos and Carvajal syndromes, rare autosomal recessive cardiocutaneous disorders, are linked to variants in the JUP and DSP genes (Protonotarios et al., 2025). In contrast, cardiac hypertrophy is not typically characterized by large-scale rearrangement of adherens junctions and desmosomes. Instead, early hypertrophic remodeling is associated with prolonged action potentials and conduction abnormalities, primarily attributed to gap junction dysfunction (Jin et al., 2010). Nevertheless, alterations in gap junction organization within intercalated discs may secondarily affect other junctional complexes. Supporting this, a study using a Wistar rat model of pressure-overload hypertrophy induced by abdominal aortic constriction reported increased expression of desmoglein-2, desmocollin-2, plakoglobin, β-catenin, and N-cadherin 90 days after surgery (dos Santos et al., 2016). In contrast, our model showed no significant changes in the expression of desmosomal or adherens junction proteins, but revealed pronounced regulation at the level of phosphorylation (Figure 6).

Although many of the identified phosphorylation sites remain functionally uncharacterized, their alterations may indicate involvement in pathological processes in SHR rats. Intriguingly, phosphorylation of adherens junction and desmosomal proteins was markedly reduced in SHR hearts. Notably, trandolapril reversed the dephosphorylation of plakophilin-4, p120-catenin, cadherin-2, desmoglein-2, and afadin, suggesting restoration of intercellular mechanical integrity. Pyridostigmine also modulated phosphorylation at specific sites, including Ser788 in cadherin-2, which is known to reduce binding to catenin δ1 and enhance cardiomyocyte adhesion (Wang et al., 2024). Additionaly, pyridostigmine also induced distinct dephosphorylation at Tyr2824 in desmoplakin (Dsp).

Phosphorylation patterns of cytoskeletal proteins also differed between SHR and WKY rats. Desmin was hyperphosphorylated at Ser31 in SHR hearts, consistent with its propensity to form amyloid-like aggregates (Rainer et al., 2018; Makihara et al., 2016). Pyridostigmine restored Ser31 phosphorylation, whereas trandolapril had only partial effects. Comparable Ser31 phosphorylation has been reported in the left ventricles of a Wistar rat model of ischemic heart failure (Bouvet et al., 2016). Both drugs restored altered phosphorylation of troponin T (Tnnt3, Tyr227), whereas phosphorylation of tropomyosin α-1 (Tpm1, Ser283) was recovered only by pyridostigmine. This may promote tropomyosin polymerization and enhance actin filament stability (Heeley et al., 1989; Sano et al., 2000; Yuan et al., 2008). Trandolapril additionally modulated phosphorylation of several sarcomeric proteins, including myosin light chain-2 (Myl2), myotilin (Myot), and troponin I (Tnni3). In particular, hyperphosphorylation of Myl2 at Ser19 may enhance contractility (Bresnick et al., 1995). Titin (Ttn), the giant sarcomeric protein spanning from the Z-disc to the M-line, exhibited site-specific phosphorylation changes. Trandolapril increased phosphorylation at Ser12995 in the PEVK region, a known target of CaMKIIδ and PKCα (Hamdani et al., 2013; Kotter et al., 2016). Although the functional consequences remain unclear, this modification may influence passive stiffness and contribute to anti-hypertrophic effects. Other titin phosphorylation sites in SHR hearts were partially restored by both treatments, reflecting their broader impact on sarcomeric mechanical properties. Obscurin, which contains a serine/threonine kinase domain (Kin1), was dephosphorylated in SHR rats and its phosphorylation was restored by both treatments (Supplementary Table S4), suggesting that it may function as a key regulator of phosphorylation in junctional and structural proteins. Pyridostigmine also induced distinct phosphorylation changes at Ser78 in myomesin-2 (Myom2), and Thr210 in myosin heavy chain 7 (Myh7). Given that pyridostigmine did not reverse hypertrophy in SHR rats (Hejnova et al., 2024), these phosphorylation events likely contribute to other cellular or tissue-level processes.

The global decrease in phosphorylation of intercellular junction and sarcomeric proteins observed in SHR hearts is unlikely to result from protein degradation, as total protein levels remained unchanged (Hejnova et al., 2024). Potential mechanisms include reduced kinase activity or relocalization, increased phosphatase activity, or diminished ATP availability. Analysis of kinase and phosphatase phosphorylation patterns revealed no major treatment-induced differences (Supplementary Table S4), suggesting that spatial compartmentalization or specific kinase–substrate interactions may underlie the observed changes.

Metabolic pathways were also affected. SHR hearts exhibited altered expression and phosphorylation of proteins involved in mitochondrial electron transport and the TCA cycle, consistent with previous reports of impaired mitochondrial function in this model (Meng et al., 2009; Tang et al., 2014). Both pyridostigmine and trandolapril restored the levels of respiratory chain components, assembly factors, and TCA cycle enzymes, with trandolapril showing greater efficacy and selectivity. Changes in the phosphorylation of acetyl-CoA carboxylases (Acaca, Acacb), pyruvate dehydrogenase (Pdha1), and related enzymes indicate modulation of energy metabolism, potentially improving ATP availability. Given the reported ATP deficiency in hypertrophic SHR hearts (Shimamoto et al., 1982; Li, 2014), these effects likely contribute to the anti-hypertrophic action of trandolapril.

Pyridostigmine elevates acetylcholine levels at the terminals of the parasympathetic nervous system, where the M2 muscarinic receptor is the predominantly expressed subtype. While M2 expression remains relatively unchanged in the hypertrophic heart, the M3 muscarinic receptor is significantly upregulated (Liu et al., 2013; Tomankova et al., 2015; Dolejsi et al., 2024). Biochemically, stimulation of M2 receptors inhibits protein kinase A (PKA) activity through Gi protein-mediated inhibition of adenylate cyclase. The resulting reduction in cAMP levels leads to the closure of L-type Ca^2+^ channels and a consequent decrease in contractile force (Dolejsi et al., 2024). As mechanical tension is reduced, desmosomes and adherens junctions are subjected to less stress, thereby diminishing the need for adaptive remodeling of these mechanical intercellular junctions. However, muscarinic signaling is complex and involves multiple downstream pathways. In addition to M2-mediated inhibition of PKA, activation of the upregulated M3 receptor stimulates protein kinase C (PKC) and enhances nitric oxide (NO) production (Dolejsi et al., 2024). In particular, the PKCα isoform has been implicated in desmosome internalization during tissue remodeling (McHarg et al., 2014). Furthermore, signaling through both receptor subtypes converges on the activation of extracellular signal-regulated kinase (ERK) (Dolejsi et al., 2024; Mao et al., 2023), while pharmacological stimulation of M2 receptors has also been shown to increase the activity of the tyrosine kinase Src (Mao et al., 2023). These downstream kinases—PKA, PKC, ERK, and Src—represent plausible candidates for the phosphorylation of structural proteins comprising desmosomes and adherens junctions, thereby contributing to the regulation of intercellular junctional remodeling.

In parallel with the enhancement of parasympathetic signaling by pyridostigmine, trandolapril modulates these structural connections through suppression of AT1 receptor-mediated signaling. This inhibition attenuates the activity of PKC, ERK, and Src kinases (Kawai et al., 2017), thereby limiting pathological cellular growth and remodeling. At the same time, trandolapril enhances signaling through bradykinin B2 receptors, leading to activation of the Akt/mTOR/nNOS pathway and stimulation of cGMP-dependent protein kinase (PKG). PKG has been shown to phosphorylate both sarcomeric proteins and key components of adherens junctions, thereby contributing to the regulation of cardiac structure and function (Boycott et al., 2020).

The functional convergence of these drug-induced kinase cascades is clearly reflected in our phosphoproteomic data. For example, plectin has been reported to be phosphorylated at Ser4642 by MNK2, a downstream effector of the ERK1/2-dependent MAPK signaling cascade, as well as by PKA (Bouameur et al., 2013). Notably, the human phosphosite Ser4642 corresponds structurally to Ser4645 in rat plectin (Supplementary Table S3). In our study, phosphorylation of plectin at Ser4645 was significantly increased following treatment with both drugs (Figure 3; Supplementary Table S2), suggesting enhanced activity of PKA and/or MNK2. These findings support the notion that therapeutic intervention promotes the coordinated activation and subcellular targeting of key signaling kinases, including PKA, ERK, and PKC, thereby modulating junction-associated substrates and contributing to the remodeling of cardiac cytoarchitecture.

In summary, our findings indicate that dysregulated phosphorylation of intercellular junction, cytoskeletal, and sarcomeric proteins contributes to hypertension-induced cardiac hypertrophy in SHR rats. Trandolapril, and to a lesser extent pyridostigmine, partially restore phosphorylation patterns, stabilizing intercellular junctions and sarcomeric structure while improving mitochondrial and metabolic function. These results underscore the critical role of phosphorylation-dependent regulation in cardiac remodeling and highlight potential therapeutic targets in hypertensive heart disease.

## 5 Limitations of the study

Although this study provides a detailed and comprehensive map of the proteomic and phosphoproteomic alterations induced under the experimental conditions, several limitations should be acknowledged. First, our conclusions are based primarily on high-throughput quantitative data and lack direct biochemical and functional validation. While omics approaches represent powerful tools for unbiased screening and hypothesis generation, the identified changes in protein expression and phosphorylation require independent verification. Specifically, key alterations in protein abundance and phosphorylation status should be validated in future studies using targeted approaches such as Western blotting, immunohistochemistry, or quantitative real-time PCR (qPCR), where appropriate. Furthermore, the predicted structural alterations, particularly those affecting cardiac cell–cell junctions, warrant direct visualization by transmission electron microscopy to assess potential morphological changes in the intercalated discs. Finally, establishing definitive causal relationships between the identified phosphosites and the observed cellular phenotype will require additional mechanistic studies. Approaches such as CRISPR/Cas9-mediated genome editing or site-directed mutagenesis of intercalated disc proteins could provide valuable insights into the functional significance of specific phosphorylation events and their contribution to cardiac remodeling.

## Data Availability

The datasets presented in this study can be found in online repositories. The names of the repository/repositories and accession number(s) can be found below: https://www.ebi.ac.uk/pride/archive/, PXD050397, https://www.ebi.ac.uk/pride/archive/, PXD076994.
